# Relationship between the population incidence of febrile convulsions in young children in Sydney, Australia and seasonal epidemics of influenza and respiratory syncytial virus, 2003-2010: a time series analysis

**DOI:** 10.1186/1471-2334-11-291

**Published:** 2011-10-26

**Authors:** Benjamin G Polkinghorne, David J Muscatello, C Raina MacIntyre, Glenda L Lawrence, Paul M Middleton, Siranda Torvaldsen

**Affiliations:** 1 Public Health Officer Training Program, New South Wales Ministry of Health, (Miller Street), North Sydney, (2059), Australia; 2 Centre for Epidemiology and Research, New South Wales Ministry of Health, (Miller Street), North Sydney, (2059), Australia; 3 School of Public Health and Community Medicine, The University of New South Wales, (High Street), Randwick, (2031), Australia; 4 Ambulance Research Institute, Ambulance Service of New South Wales, (Church Street), Rozelle, (2039), Australia

## Abstract

**Background:**

In 2010, intense focus was brought to bear on febrile convulsions in Australian children particularly in relation to influenza vaccination. Febrile convulsions are relatively common in infants and can lead to hospital admission and severe outcomes. We aimed to examine the relationships between the population incidence of febrile convulsions and influenza and respiratory syncytial virus (RSV) seasonal epidemics in children less than six years of age in Sydney Australia using routinely collected syndromic surveillance data and to assess the feasibility of using this data to predict increases in population rates of febrile convulsions.

**Methods:**

Using two readily available sources of routinely collected administrative data; the NSW Emergency Department (ED) patient management database (1 January 2003 - 30 April 2010) and the Ambulance NSW dispatch database (1 July 2006 - 30 April 2010), we used semi-parametric generalized additive models (GAM) to determine the association between the population incidence rate of ED presentations and urgent ambulance dispatches for 'convulsions', and the population incidence rate of ED presentations for 'influenza-like illness' (ILI) and 'bronchiolitis' - proxy measures of influenza and RSV circulation, respectively.

**Results:**

During the study period, when the weekly all-age population incidence of ED presentations for ILI increased by 1/100,000, the 0 to 6 year-old population incidence of ED presentations for convulsions increased by 6.7/100,000 (P < 0.0001) and that of ambulance calls for convulsions increased by 3.2/100,000 (P < 0.0001). The increase in convulsions occurred one week earlier relative to the ED increase in ILI. The relationship was weaker during the epidemic of pandemic (H1N1) 2009 influenza virus.

When the 0 to 3 year-old population incidence of ED presentations for bronchiolitis increased by 1/100,000, the 0 to 6 year-old population incidence of ED presentations for convulsions increased by 0.01/100,000 (P < 0.01). We did not find a meaningful and statistically significant association between bronchiolitis and ambulance calls for convulsions.

**Conclusions:**

Influenza seasonal epidemics are associated with a substantial and statistically significant increase in the population incidence of hospital attendances and ambulance dispatches for reported febrile convulsions in young children. Monitoring syndromic ED and ambulance data facilitates rapid surveillance of reported febrile convulsions at a population level.

## Background

Febrile convulsions (or febrile seizures) are defined as fever combined with convulsion in children aged 6 months to 5 years, without underlying central nervous system disease. They are relatively common, are generally self-limiting, and peak incidence is seen in children aged 18 months [1,2]. In a recent prospective population-based study in Finland, the estimated incidence rate up to the age of four years was 6.9% [3]. Febrile convulsions can lead to severe outcomes, with approximately 25% of cases admitted to hospital, although death is rare [4,5].

Among respiratory pathogens, influenza and respiratory syncytial virus (RSV) are responsible for the highest burden of hospitalisation of young children in Australia [6]. Influenza virus is responsible for annual epidemics of varying severity, and in an average year 20% of all children will be infected with influenza [7]. RSV infection is the most important respiratory pathogen of early childhood commonly causing severe bronchiolitis and pneumonia requiring hospital admission [8,9]. By 2 years of age, most children have been infected with RSV and 1-3% of infections require hospitalisation [10]. RSV and influenza co-circulate and can manifest similar symptoms including fever. Epidemics of each virus occur yearly during winter and early spring in temperate climates [7,11].

Influenza infection is recognized as an important cause of febrile convulsions in young children [12,13]. RSV infections can also be complicated by convulsions, but much less frequently than influenza infections [4,14-16]. However, a febrile convulsion can be triggered by any cause of fever, including other viral infections such as human herpesvirus 6, adenovirus, parainfluenza and rotavirus [4,17]. Live viral vaccines can also cause febrile convulsions; for example, the measles, mumps and rubella vaccine has been associated globally with febrile convulsions within 2 weeks of vaccination [18,19].

Investigating the relationship between the population incidence of febrile convulsions associated with natural infection by common seasonal respiratory illnesses such as influenza and respiratory syncytial virus (RSV) can inform debate on the risks and benefits of preventive activities such as immunisation which has recently come under scrutiny as a potential cause of febrile convulsions. In April 2010, health authorities in the state of Western Australia (WA) reported that children < 5 years of age were experiencing high rates of febrile convulsions following vaccination with the 2010 southern hemisphere seasonal trivalent influenza vaccine - approximately nine febrile convulsions per 1000 seasonal vaccine doses administered in WA [20]. An ongoing national evaluation [21] was then initiated by the Therapeutic Goods Administration (TGA), Australia's regulatory body for medicines, medical devices and blood and tissue products.

New South Wales (NSW), Australia has a population of approximately 7.25 million people. The population predominantly resides on the eastern seaboard and approximately 4.58 million people live in the state's capital city Sydney. The NSW Department of Health (which became the NSW Ministry of Health after the study period) monitors the daily and weekly incidence of convulsions through established syndromic surveillance of emergency department (ED) presentations [22] and ambulance dispatches. We aimed to use routinely collected ED and ambulance syndromic surveillance data to determine whether the seasonal peaks in the population incidence of febrile convulsions in children are associated with seasonal epidemics of either influenza or RSV and whether these data constituted a feasible method of rapid population surveillance for febrile convulsions.

## Methods

### Data Sources

Emergency department (ED) data were obtained from the NSW ED data collection on the Health Outcomes and Information Statistical Toolkit (HOIST) database held at the NSW Department of Health, only for the 35 public hospital EDs in the greater Sydney metropolitan area. Public hospitals provide almost all ED services in Sydney, as there are only two private hospitals with emergency departments. Ambulance dispatch data were obtained from the Ambulance Service of NSW computer-aided dispatch (CAD) database for Sydney. ED data for the period 1 January 2003 - 30 April 2010 were used, but ambulance dispatch data were only available for the period 1 July 2006 - 30 April 2010.

ED presentations were selected using International Classification of Diseases (ICD) codes from the clinical modification of the ninth revision (ICD-9-CM) and the Australian modification of the tenth revision (ICD-10-AM) and the equivalent Systematized Nomenclature of Medicine - Clinical Terminology (SNOMED-CT) concepts. ICD-9-CM codes selected were: 466.1, 487, 779.0, and 780.3. ICD-10-AM codes selected were: J10-J11, J21, P90 and R56A. A list of SNOMED-CT concepts selected is available from the corresponding author. The range of diagnoses chosen did not include specific diagnoses of epilepsy or epileptic seizures.

The databases used for this study did not include patient names, addresses, or dates of birth, and ethics approval was therefore not required.

### Descriptive analysis

For the 35 Sydney public hospitals combined, we prepared a weekly count time series of presentations that had a provisional ED diagnosis of convulsions. Only presentations for children aged < 6 years in the Sydney region were included. We also prepared a weekly count time series of urgent ambulance dispatches for children aged < 6 years that were assigned a dispatch problem category of 'fitting or convulsions' during the emergency telephone call. Despite the definition of febrile convulsions occurring in children 6 months or older, we were unable to retrieve a population estimate for children aged 6 months to 1 year, thus all children < 1 year were included. This may mean our population incidence rates are slightly underestimated.

To indicate periods of influenza and RSV circulation, we prepared weekly count time series of all-age ED presentations assigned a diagnosis of influenza-like illness (ILI) and ED presentations assigned a diagnosis of bronchiolitis for children aged < 3 years. We chose to limit bronchiolitis in this analysis to children less than 3 years of age because more than 96% of all ED presentations for bronchiolitis in the dataset studied occurred in children in this age group, whereas influenza-like illness was more evenly distributed across age groups. In south-eastern Sydney, in a time period overlapping this study, Schindeler and colleagues found a large, significant, and independent association between ED presentations for influenza and positive laboratory tests for influenza viruses and between ED presentations for bronchiolitis, and RSV laboratory counts [23]. In an earlier overlapping time series, Zheng and colleagues also demonstrated the association between the ED influenza-like syndrome and laboratory specimens positive for influenza for 49 EDs across NSW [24].

**Figure 1:**
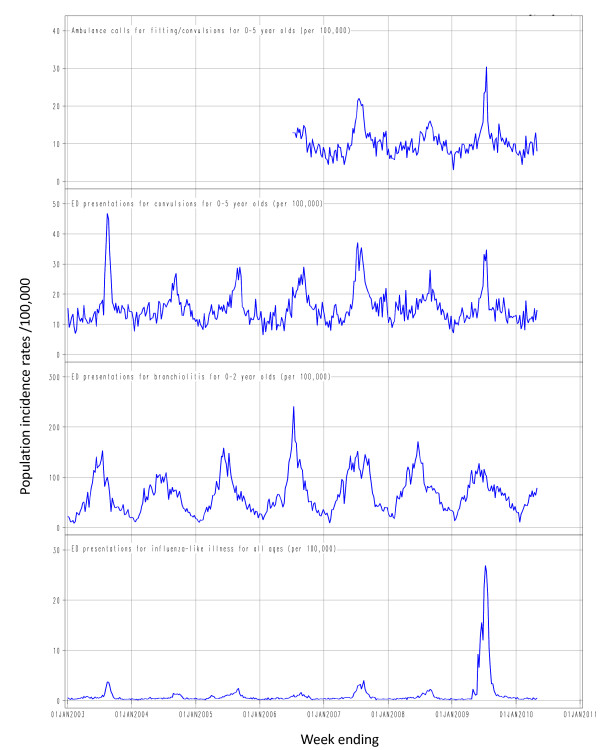
**Weekly population incidence rates of Emergency Department (ED) presentations and urgent ambulance dispatches^1 ^for selected syndromes: 1 January 2003 to 30 April 2010**. ^1^Electronic Ambulance dispatch data was only available from July 2006.

Weekly population rates for each time series were calculated using a denominator of weekly population estimates obtained by interpolating between mid-year estimates for the Sydney region. The time series were then graphed (Figure 1) and the graphs were visually inspected to compare the incidence of convulsions with the circulation of influenza and RSV.

### Statistical analysis

To further examine these relationships, a semi-parametric generalized additive models (GAM) analysis was used to determine the association between the population incidence rates of ED presentations for convulsions and ambulance calls for fitting or convulsions for children < 6 years old and the population incidence rates of ED presentations for bronchiolitis in children < 3 years old and for influenza-like illness (ILI) in persons of all ages. Using a natural cubic smoothing spline curve fitted non-parametrically to the outcome time series, the GAM analysis controlled the autocorrelation, or non-independence of model residuals which can arise in analysis of time series that contain seasonality and trend. This has the effect of allowing other independent variables to be examined for their short-term associations with the outcome time series, in this case weekly counts of ED or ambulance convulsions. The general method used is outlined in greater detail in a previous study [23].

Because we used rates per 100,000 population for our dependent variables, which take on continuous values, we assumed the model residuals would follow a normal distribution. We constructed separate models: one for the ED convulsions outcome, and one for the ambulance fitting and convulsions outcome.

Prior to fitting the final models for each outcome, we separately evaluated different lags in the relationship between the ILI series and the outcomes and the bronchiolitis series and the outcomes. This was done because it is possible that the timing of illness patterns captured by each ED and ambulance variable we used may vary in response to bronchiolitis and RSV epidemics. Lags were evaluated by separately fitting the single variable models for bronchiolitis and ILI for the 5 lags from -2 and +2 weeks to determine which had the best model fit according to model deviance and the strength of the parameter estimate.

The final model was:

$$Expected\left(Y_{t}\right)=\beta_{0}+\beta_{1}bronchiolitis+\beta_{2}ILI+\beta_{3}Year2009+\beta_{4}\left(Year2009xILI\right)+S\left(time\right)$$

Where Y_t _is the weekly population rate of ED convulsions or ambulance calls for fitting and convulsions; β_1 _is the parameter estimate of the linear association between the bronchiolitis ED presentation rate and a 1/100,000 increase in the outcome rate; and β_2 _is the parameter estimate of the linear association between ILI ED presentation rates and a 1/100,000 increase in the outcome rates. Year2009 was an indicator variable having the value 1 for all weeks in 2009 and 0 for all other weeks. This was included because it was evident from inspection of the time series that the relative scale of peaks in convulsions activity did not match the scale of peaks in ILI activity in EDs. The interaction between Year2009 and ILI was included to allow the relationship between ILI and the outcome to differ in 2009. S(time) is the smoothing spline for time in weeks. We aimed to use 4 degrees of freedom per year for the spline. That is, the smoothing curve had 4 segments per year. This was sufficient to remove residual autocorrelation in our previous work [23].

For the initial lag analysis for ILI versus convulsions, we included the Year2009 variable and its interaction with ED ILI as this dramatically improved model fit. Splines were included in these models as well to control autocorrelation. Because the lag analysis for bronchiolitis was not able to control adequately for autocorrelation, we used a spline with 8 degrees of freedom per year in those models.

The GAM analysis was performed using the GAM procedure in SAS version 9.2. The only option used in the model statement for PROC GAM was DIST = NORM for a normally distributed response variable. Autocorrelation was assessed by visual inspection of autocorrelation plots generated using the ARIMA procedure. Normality of model residuals was evaluated by visual inspection of Q-Q plots.

## Results

Over the period 1 January 2003 to 30 April 2010 there was a total of 18,405 presentations to Sydney EDs assigned a provisional diagnosis of convulsions in children aged < 6 years old. This equates to an average incidence rate in this age group of 819/100,000 population per year. Over the period 1 July 2006 to 30 April 2010 there was a total of 6602 emergency ambulance dispatches for fitting or convulsions in children < 6 years old. This equates to an average incidence rate in this age group of 583/100,000 population per year.

The time series for convulsions in both the ambulance and ED series (Figure 1) follow broadly similar seasonal trends. Clear peaks occurred in the ED series during 2003, 2007 and 2009, all years that experienced higher than usual influenza activity [25-27]. Convulsion rates in those years rose to peaks of 47, 36 and 35 per 100,000 per week, respectively. Peaks at similar dates are evident in the ambulance series in 2007 (22/100,000 population/week) and 2009 (30/100,000 population/week). The peaks in convulsions in < 6 year olds for both the ED and ambulance series clearly coincide with peaks in ED presentations for influenza-like illness. However, the magnitude of the relationship is clearly lower in 2009 than in previous years (Figure 1).

Although the rates in the shorter ambulance series were consistently lower than in the ED series, the pattern of ambulance dispatches for fitting and convulsions was consistent over time with the ED series, except during the 2009 pandemic influenza season where the ratio of ambulance dispatches to ED presentations increased (Figure 1).

While there is some seasonal pattern in the convulsion series outside of influenza seasons, peaks in bronchiolitis activity do not coincide with significant peaks in convulsions (Figure 1).

RSV circulation as indicated by ED bronchiolitis presentations did not appear to be associated with changes in rates of convulsions. The highest seasonal peak in ED bronchiolitis presentations was 353 ED presentations per week in July 2006 and did not appear to correspond to any peaks in febrile convulsions in either the ED or ambulance time series.

### Time series analysis: evaluation of lagged relationships

For all models that included ED ILI, the parameter estimate for the interaction between ILI and convulsions in 2009 was found to be strongly negative indicating a weaker association in 2009 between ED ILI presentations and febrile convulsions.

The ILI time series were most strongly associated with ED convulsions and ambulance fitting and convulsions at lag -1 week. The same was found for the bronchiolitis time series and ED convulsions. Lag -1 week represents a situation where the changing incidence of convulsions precedes the changing incidence of ILI and bronchiolitis by 1 week. The parameter estimates for lag 0 were very similar to those for lag -1. While there was a statistically significant positive association at lag +2 for the bronchiolitis and ambulance fitting and convulsions series, considerable autocorrelation remained in the model residuals, so we concluded that there was not a clear relationship found for that model (Table 1).

**Table 1:** Lag analysis: change in incidence rates/100,000 population for Ambulance and ED convulsions in children < 6 years associated with a 1/100,000 population increase in all ages ED influenza-like illness and bronchiolitis in children < 3 years

Independent Variable	Lag^1 ^(weeks)	Ambulance Convulsions		ED Convulsions	
		
		Parameter estimate (95% CI)	P-value	Parameter estimate (95% CI)	P-value
	-2	2.907 (2.374-3.440)	<0.0001	5.119 (4.535-5.704)	<0.0001
ED^2^	-1	3.240 (2.769-3.711)	<0.0001	6.678 (6.181-7.175)	<0.0001
Influenza-	0	2.948 (2.483-3.412)	<0.0001	6.597 (6.096-7.099)	<0.0001
like illness	+1	0.122 (-0.065-0.309)	0.203	0.546 (0.247-0.844)	0.0004
	+2	-0.013 (-0.143-0.118)	0.8483	0.083 (-0.132-0.298)	0.4499

	-2	-0.007 (-0.014-0.000)	0.0631	-0.008 (-0.016--0.001)	0.033
ED^3^	-1	0.005 (-0.002-0.012)	0.1559	0.023 (0.015-0.030)	<0.0001
Bronchiolitis	0	0.002 (-0.005-0.009)	0.5969	0.020 (0.013-0.028)	<0.0001
	+1	0.002 (-0.005-0.008)	0.6648	-0.006 (-0.014-0.002)	0.1187
	+2	0.011 (0.004-0.018)	0.0017	-0.010 (-0.017--0.002)	0.013

^1^A negative lag refers to a change in convulsions incidence occurring prior to the change in ILI or bronchiolitis ED visits. A positive lag indicates convulsions incidence changes after the change in ILI or bronchiolitis.

^2^To control for trend and seasonality, a natural cubic spline with 4 degrees of freedom per year was used.

^3^To control for trend and seasonality, a natural cubic spline with 4 degrees of freedom per year was insufficient, so 8 degrees of freedom were used.

### Statistical analysis: final models

We then fitted the final model for each outcome, ED and ambulance convulsions. From the results of the lag analysis, we included a lag of -1 in these models. That is, the ED and ambulance convulsions were shifted one week earlier relative to the ED ILI and bronchiolitis time series.

Controlling for trend, seasonality, the 2009 pandemic influenza year and bronchiolitis, when the incidence rate of ED presentations for ILI in persons of all ages increased by 1/100,000 population in a week, the incidence rate of ED presentations for convulsions in children under 6 years increased by approximately 6.7/100,000 population one week earlier. The incidence rate of ambulance calls for fitting and convulsions in children under 6 years increased by 3.2/100,000. Controlling for trend, seasonality, the 2009 pandemic influenza year and ILI, when the incidence rate of ED presentations for bronchiolitis in children under three years increased by 1/100,000 population, the incidence rate of ED presentations for convulsions in children under 6 years increased by 0.01/100,000 one week earlier. We did not find a statistically significant independent association between bronchiolitis and ambulance fitting and convulsions (Table 2).

**Table 2:** Final model: change in incidence rates/100,000 population for Ambulance and ED convulsions in children < 6 years, associated with a 1/100,000 population increase in all ages ED influenza-like illness and bronchiolitis in children < 3 years

Dependent variable^1^	Independent variable	Parameter estimate (95%CI)	P-value
Ambulance Convulsions (1 Jul 2006 - 30 April 2010)	ED Influenza-like illness	3.24 (2.69-3.78)	<0.0001
	
	ED Bronchiolitis	0.00 (0.00-0.01)	0.2681

ED Convulsions (1 Jan 2003 - 30 April 2010)	ED Influenza-like illness	6.66 (6.11-7.21)	<0.0001
	
	ED Bronchiolitis	0.01 (0.00-0.02)	0.0067

^1^ED and ambulance convulsions series were shifted one week earlier than the ILI and bronchiolitis series (lag = -1).

**Figure 2:**
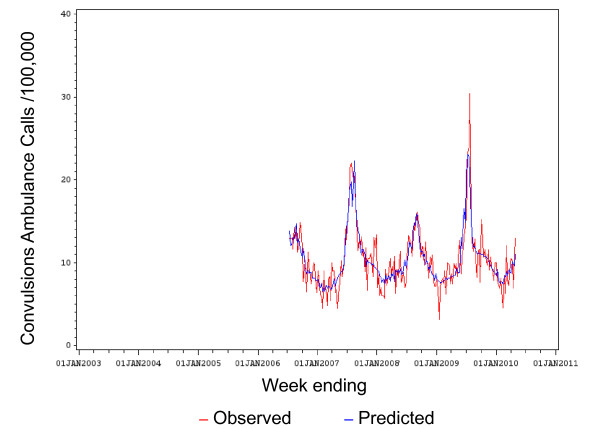
**Observed versus predicted population rates/100,000 for convulsions ambulance calls by week: 1 Jul 2006 to 30 April 2010**.

**Figure 3:**
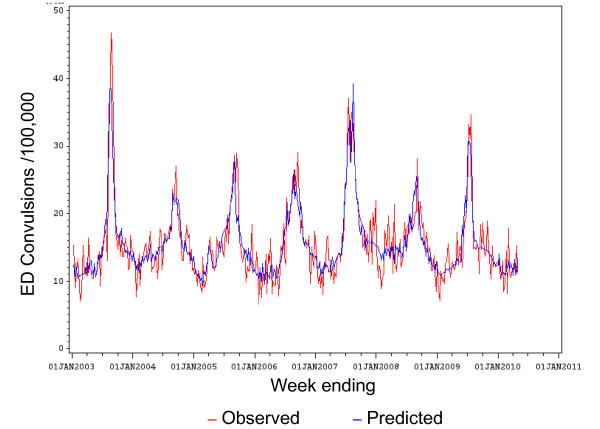
**Observed versus predicted population rates/100,000 for convulsions ED presentations by week: 1 Jan 2003 to 30 April 2010**.

The observed versus the fitted (predicted) values of population rates for convulsions ambulance calls are shown in Figure 2. The observed versus the fitted (predicted) values of population rates for ED convulsions are shown in Figure 3.

## Discussion

Using population data over seven consecutive influenza seasons including the 2009 NSW winter epidemic of pandemic (H1N1) 2009 influenza (pH1N1), we found a large, significant and independent association between the population incidence rate of ED presentations for ILI syndrome and the population incidence rate of ED presentations for convulsions. This is consistent with previous international studies using hospital inpatient and general practice data [4,10,13]. We also saw a smaller, yet significant and independent association between the population incidence rate of ED presentations for ILI syndrome and the population incidence rate of ambulance calls for fitting and convulsions. The relationship between ED bronchiolitis and ED convulsions was independent and statistically significant, yet very small. We did not find a statistically significant association between ED bronchiolitis and ambulance calls for fitting and convulsions. This may be partly due to the shorter ambulance time series.

Ambulance data are an alternative source of rapid surveillance which have not been widely used in public health practice previously, and our study allows evaluation of this novel source of data. We found routinely collected ED and ambulance dispatch time series data useful tools for rapidly estimating the population level impact of seasonal respiratory virus epidemics on incidence of febrile convulsions in young children. These data sources should be considered in public health surveillance in situations where they can be timelier than other commonly used data sources, such as laboratory notifications.

The lag analysis did not demonstrate a strong distinction between lag 0 weeks and lag -1 week. Since the ILI, bronchiolitis, and convulsions time series are likely to be responding at least in part to actual influenza and RSV epidemics in the community, the disease outcomes such as convulsions should occur at the same time. However, while influenza and bronchiolitis are acute illnesses, the epidemics may move through different age groups at slightly different times or rates. Also, the stage of the natural course of illness at which febrile convulsions occurs may differ from the stage when a person experiencing ILI or bronchiolitis would seek medical attention. The similarity of the results of the lag analysis when lagging or not lagging by -1 shows that this timing difference may not be important and may represent limitations in the precision of the data sources.

Two previous NSW studies using the Emergency Department Data Collection (EDDC) with time series that overlap that used in this study have demonstrated that ED ILI and bronchiolitis syndromes constitute reliable proxies for the circulation of influenza and RSV in NSW [23,24]. However, there are limitations to this syndromic data. The ED diagnoses and ambulance dispatch problem categories are 'syndromic', that is, they include all fits or convulsions of any cause. Thus, both the ED and ambulance time series include a proportion of non-febrile convulsions that are unrelated to respiratory illness. These may include early epileptic seizures or convulsions due to electrolyte imbalances [2]. Also, from these datasets we are unable to report on the number of recurrent febrile convulsions or subsequent sequelae after the initial emergency presentation or ambulance transport. The recording of diagnoses or problems in the EDDC is completed by clinicians or clerical staff, not by trained coders, so variation in coding practices can occur. Some cases of febrile convulsions could be assigned an alternative preliminary ED diagnosis or ambulance dispatch reason code, such as "fever" or "unspecified infection". Similar alternate preliminary diagnoses could be assigned to presentations for influenza-like illness and bronchiolitis.

The results of a population based time series study [4] conducted in the Dutch province of Friesland between April 1998 and April 2002 suggested an association between febrile convulsions and influenza at the population level. Febrile convulsions in children aged between 3 months and 5 years were reported by participating general practitioners using standard notification cards mailed every 3 months. Poisson regression analysis showed clear concordances between convulsions peaks and influenza peaks. As in our study, RSV did not appear to be associated with convulsions. An earlier study in Hong Kong of children hospitalized with an influenza infection in 1997 and 1998, found that almost one-fifth of the children suffered a febrile convulsion [13]. However, given that only approximately one quarter of children who suffer febrile convulsions are admitted to hospital [4], our study of emergency department presentations and ambulance calls for convulsions may better capture the majority of febrile convulsion episodes.

The epidemic of pH1N1 virus in Sydney in 2009 was associated with a thousandfold increase in all-age ED presentations for ILI above baseline and an extended epidemic period as shown in Figure 1. Yet in 2009, rates of ED convulsions presentations for children aged <6 years presenting to NSW EDs did not rise as high as the 2003 or 2007 influenza season peaks. While we do not have ambulance data for 2003, the 2009 ambulance convulsions peak was higher than the 2007 peak (Figure 1). It is reasonable to assume that the reported numbers for ED febrile convulsions and ambulance fitting and convulsions are accurate as the symptoms of a convulsion are hard to mistake and unlikely to be misinterpreted due to anxiety. How then can we explain these contradictory relationships between ED ILI presentations and febrile convulsions compared with previous years?

The predominant influenza strain in NSW during 2003 was the emergent A/Fujian/411/2002 (H3N2)-like virus, which led to widespread severe outbreaks globally [25]. In 2007, A/Brisbane/10/2007 (H3N2)-like and A/Solomon Islands/3/2006 (H1N1)-like viruses were co-circulating, causing severe illness in young children in Australia, including six deaths [26]. This contrasts with the 2009 influenza season which was dominated by A/California/7/2009 (H1N1)-like influenza (pH1N1) which was characterized by a very high attack rate, particularly in children, but was only moderately virulent [27].

By controlling for the 2009 pandemic year in our model, we found that the relationship between ED ILI presentations and both ED and ambulance convulsions was much weaker in 2009. This may be due to a number of factors. ED presentation rates may have been increased by the high media coverage of the "swine flu pandemic", healthy people attending EDs seeking "swine flu free" certification to avoid exclusion from work or school and people with influenza symptoms who were directed to attend an ED or hospital influenza clinic rather than a general practice. Many of these influenza clinic presentations were recorded as ED presentations [28]. Changes in diagnostic behaviour may also have led to more patients receiving an ILI diagnosis than usual. The relatively mild nature of the majority of pandemic influenza infections in 2009 compared with seasonal influenza infections could explain the disparity in the relationship with febrile convulsions in 2009 [25,29].

The apparently stronger relationship in 2009 for ambulance convulsions calls compared with ED convulsions in the same year perhaps indicates that parents, schools and childcare services had a lower tolerance for symptoms of fitting and convulsions in children during the pandemic, and thus were more likely to request an ambulance.

During the time period studied, detailed records on influenza vaccinations of children were not routinely collected in NSW, therefore we were unable to make a direct comparison between vaccination rates and febrile convulsions rates. Prior to 2010, government funded seasonal vaccination eligibility was limited to certain high risk groups, and thus vaccination rates in NSW children were traditionally low particularly in comparison with WA which has had a childhood influenza vaccination programme since 2008 [30]. Eligibility for subsidized influenza vaccine was extended in 2010 and, due to the ongoing pandemic, vaccination was extensively promoted.

In December 2009, the Australian government funded Panvax^® ^H1N1 Vaccine Junior (Panvax Junior) was released. NSW Health records indicate that over 140,000 single dose vials of Panvax Junior were distributed in NSW between December 2009 and April 2010. Additionally, 17,420 doses of the Australian Government funded trivalent Fluvax^® ^Junior were distributed in NSW between March and April 2010. The NSW Population Health Survey programme conducts Computer Aided Telephone Interviews (CATI) with NSW residents and since February 2010 has reported on pH1N1 influenza vaccine coverage of children aged 0-9 years. The unweighted coverage estimate in this age group for April 2010 was 19% [31]. Despite this apparent increase in influenza vaccination coverage in NSW children between December 2009 and April 2010, Figure 1 does not indicate a corresponding upward trend in febrile convulsions over the same period.

## Conclusions

This study demonstrates that influenza epidemics, and not RSV epidemics, are associated with substantial increases in febrile convulsions at the population level in young children in Sydney, Australia. It also provides evidence that monitoring ED and ambulance dispatch data is a feasible method for conducting rapid surveillance of febrile convulsions. In the light of these findings, recent concern about a reported eightfold increase in the risk of convulsions in children following administration of the 2010 seasonal influenza vaccine must be evaluated with the recognition that natural influenza infections may be responsible for a substantial incidence of febrile convulsions in young children. These outcomes of natural infection may confound the attribution of febrile convulsions to vaccination.

## Competing interests

The authors declare that they have no competing interests.

## Authors' contributions

BP conceived of the study, conducted the descriptive analysis and drafted the paper. DM devised and conducted the statistical analysis and assisted with developing the study and reviewing revisions of the paper. RM, GL and PM contributed to the development of the study and the revision of the paper. ST assisted with the development and revision of the paper. All authors read and approved the final manuscript.

## Pre-publication history

The pre-publication history for this paper can be accessed here:

http://www.biomedcentral.com/1471-2334/11/291/prepub
